# Identification of two key genes controlling chill haze stability of beer in barley (*Hordeum vulgare* L)

**DOI:** 10.1186/s12864-015-1683-1

**Published:** 2015-06-11

**Authors:** Lingzhen Ye, Yuqing Huang, Fei Dai, Huajiang Ning, Chengdao Li, Meixue Zhou, Guoping Zhang

**Affiliations:** Agronomy Department, Zhejiang University, Hangzhou, 310058 People’s Republic of China; Department of Agriculture and Food, Western Australia 3 Baron-Hay Court, South, Perth, Australia; Tasmanian Institute of Agriculture, University of Tasmania, P.O.Box 46, Kings Meadows, TAS 7249 Hobart, Australia

**Keywords:** Barley, Beer haze, Malt, Quantitative trait loci (QTL)

## Abstract

**Background:**

In bright beer, haze formation is a serious quality problem, degrading beer quality and reducing its shelf life. The quality of barley (*Hordeum vulgare* L) malt, as the main raw material for beer brewing, largely affects the colloidal stability of beer.

**Results:**

In this study, the genetic mechanism of the factors affecting beer haze stability in barley was studied. Quantitative trait loci (QTL) analysis of alcohol chill haze (ACH) in beer was carried out using a Franklin/Yerong double haploid (DH) population. One QTL, named as *qACH,* was detected for ACH, and it was located on the position of about 108 cM in chromosome 4H and can explain about 20 % of the phenotypic variation. Two key haze active proteins, BATI-CMb and BATI-CMd were identified by proteomics analysis. Bioinformatics analysis showed that *BATI-CMb* and *BATI-CMd* had the same position as *qACH* in the chromosome. It may be deduced that *BATI-CMb* and *BATI-CMd* are candidate genes for *qACH*, controlling colloidal stability of beer. Polymorphism comparison between Yerong and Franklin in the nucleotide and amino acid sequence of *BATI-CMb* and *BATI-CMd* detected the corresponding gene specific markers, which could be used in marker-assisted selection for malt barley breeding.

**Conclusions:**

We identified a novel QTL, *qACH* controlling chill haze of beer, and two key haze active proteins, BATI-CMb and BATI-CMd. And further analysis showed that *BATI-CMb* and *BATI-CMd* might be the candidate genes associated with beer chill haze.

**Electronic supplementary material:**

The online version of this article (doi:10.1186/s12864-015-1683-1) contains supplementary material, which is available to authorized users.

## Background

Beer is one of the oldest and also most widely consumed alcoholic beverages, and it is commonly produced from malt barley as main raw material. Haze is often developed during beer storage or transportation, resulting in reduced shelf life and degraded quality of beer. Beer haze can be divided into biological and non-biological ones. The biological haze can be avoided or reduced during beer processing, as it is caused by the wild yeast or bacteria due to poor hygiene. In contrast, the non-biological haze is not easy to be dealt with, because it is derived from brewing raw materials, such as malt barley.

The most common non-biological haze is attributed to interactions between haze active proteins and certain polyphenols [1–3]. Moreover, non-biological haze is commonly divided into chill haze and permanent haze. Chill haze is formed when beer is chilled to 0 °C and it may re-dissolve when the beer is warmed to 20 °C or more, while permanent haze will remain in beer even at higher temperature. In fact, chill haze is a precursor of permanent haze, so understanding of non-biological haze formation in beer should be started from chill haze.

Some technical approaches have been available for reducing haze formation in beer, such as silica [4, 5] and polyvinylpolypyrrolidone (PVPP) adsorbent [6] treatments, but these treatments will increase the cost of beer production and deteriorate some flavor due to reduced relevant proteins, such as foam active protein. As non-biological haze formation is closely related to malt barley, the one of most efficient ways for controlling colloidal haze formation is development of the malt barley cultivars with lower content of haze-related proteins.

Beer contains lots of barley and yeast proteins, which affect beer haze stability. Many barley proteins, including hordeins [1], dimeric α-amylase inhibitor (BDAI-1) [7], CMb component of tetrameric α-amylase inhibitor (CMb) [7] and trypsin inhibitor CMe precursor (BTI-CMe) [8–11] have been considered as the haze active proteins. However, the main factors that act as the dominant role in beer haze formation are not clearly known. Meanwhile, although a great number of researches have been done on genetics and relevant genes or molecular markers of many malt quality traits, such as diastatic power [12, 13], seed dormancy [14], and protein content [12, 13], very few reports could be found about genetic controlling of haze active proteins.

In this study, we identified a QTL controlling haze formation in beer through comparing the difference in haze formation among 177 lines of a double-haploid (DH) population as well as the two parents, and found two haze active proteins through proteomics analysis. In addition, the mechanism in genotypic difference of haze formation was also proposed.

## Methods

### Plant materials and field trial

A double haploid (DH) population consisting of 177 lines, derived from a cross between Franklin and Yerong was used in this study. The field experiments were conducted in two growing seasons of 2009–2010 and 2010–2011 on the experimental farm of Zhejiang University (Huajiachi campus, Hangzhou, China). All DH lines and the two parents were sown in early November with adjacent plots in a field and each plot consisted of 10 rows with 2 m length and 0.25 m between rows [15]. In each row 50 seeds were sown. Other field managements, including fertilization, weed and disease control, were the same as applied locally. At maturity, the grains in 8 middle rows of each plot were harvested and stored in a refrigerator at 4°Cfor further measurements.

### Preparation of beer samples

The grains of each line and parents of the DH population were micro-malted according to Cai et al. [16] and micro-brewed according to Stewart et al. [17] with some modification. The procedures were briefly as follows: 200 g grain sample was micro-malted by Joe White malting system; and then 50 g of malt grist (ground by a Buhler Miag mill) with three replications was mashed in a temperature-controlled mash bath according to a European Brewery Convention (EBC) method. Water was added to a final weight of 450 g (grist/water ratio 1:8, the wort concentration was about 8.3 Bx) after finishing the mash process, and then wort was filtered by a filter paper. After adjusting the pH of the wort to 5.4 with 1 N H_3_PO_4_, the wort was sterilized at 105 °C for 30 min without hops; Then commercial beer dry yeast (JJB, the UK) was incubated with wort at a ratio of 0.6‰. The wort was fermented at a constant temperature of 8 °C for 13 days. Finally, beer was filtered by a sheet filter and bottled for further analysis.

### Trait assay

The alcohol-chill test was conducted to predict the colloidal stability of beer according to Chapon [18]. The procedures were as follows: 5 % pure ethanol was added into beer sample and carefully mixed, frozen at −8 °C for 40 min, then measured by a turbimeter (HANNA HI93124). In this study, EBC unit was used for alcohol-chill haze degree (ACHD), characterizing the beer status at racking or during aging [19, 20].

### QTL analysis

The statistical analysis of phenotypic data, including variance and correlation analysis of ACHD in two growing seasons were accomplished using SPSS 13.0. The genetic linkage map was constructed using 496 Diversity Array Technology (DArT) and 28 microsatellite markers by software Jionmap 4 [21]. QTLs were analyzed using software MapQTL5.0 [22]. Firstly, interval mapping (IM) was done in QTL analysis, and then the closest marker with highest logarithm of the odds (LOD) score was selected as a cofactor for testing multiple QTL model (MQM). A threshold LOD > 3 was used to prove the presence of a QTL.

### Preparation of protein samples

The alcohol chill treated beers were centrifuged at 15000 g for 20 min at 0 °C. Turbid sediments and clear supernatants were then collected as ACH and control, respectively.

### Proteomics analysis

After mixed with protein loading buffer, the collected control and ACH samples were treated at 100 °C for 5 min. Then Tricine-SDS-PAGE was carried out as described by Schagger [23]. The gels were stained with Coomassie blue G250. The special protein band of about 15 kDa was excised and digested by trypsin (Promega V5280) for High performance liquid chromatography-tandem mass spectrometry (LC-MS/MS) analysis.

LC-MS/MS analysis of the digested proteins was performed using a Thermo Scientific Surveyor Plus HPLC system coupled to a Thermo Electron LTQ-Orbitrap mass spectrometer. Chromatographic separations were conducted on a reverse-phase capillary column (100 μmi.d., 10 cm long, 3 μm resin from Michrom Bioresources, Auburn, CA) with a mobile phase A of solution containing 0.1 % formic acid, 2 % acetonitrile and a mobile phase B of acetonitrile containing 0.1 % formic acid at a flow rate of 300 nL/min. The gradient was gradually increased from 5 % to 35 % of solvent B (0.1 % formic acid/ACN) within 120 min. The mass analysis was performed in a positive ionization mode. The operation conditions were as follows: ionspray voltage, 1.85 kV; source temperature, 220 °C; resolution ratio, 60000; and scanning scope, 400 ~ 2000 Da. For data processing, MASCOT search program (http://www.matrixscience.com/) was used with significant threshold of 0.05.

### DNA extraction, PCR amplification, and sequencing

Total genomic DNA was extracted from the seedlings of Franklin, Yerong and all DH lines using Genomic DNA Extraction Kit (QIAGEN) following the manufacturer’s instructions. For cloning and sequencing *MLOC_12143.1* and *MLOC_65022.1* (encoding BATI-CMb and BATI-CMd) of Yerong and Franklin, two sets of primers (Additional file 1: Table S1) were designed according to the reference sequence from barley whole genome sequence [24]. The polymerase chain reaction (PCR) was conducted using LA Taq polymerase (TaKaRa), with annealing temperature of 62 °C and 32 cycles. After purification, the PCR products were transferred into the pGEM-T Easy Vector (Promega) following the kit’s instruction. Then 20 positive clones of each gene were selected and sequenced.

For screening of gene specific markers, primers were designed by software Geneious 4.8.3 according to the insertion and deletion (InDel) polymorphism between Yerong and Franklin (Additional file 1: Table S1). The PCR products were analyzed using 2.5 % agarose gel electrophoresis.

## Results

### Phenotypic variation among the lines of Franklin/Yerong DH population

**Fig. 1:**
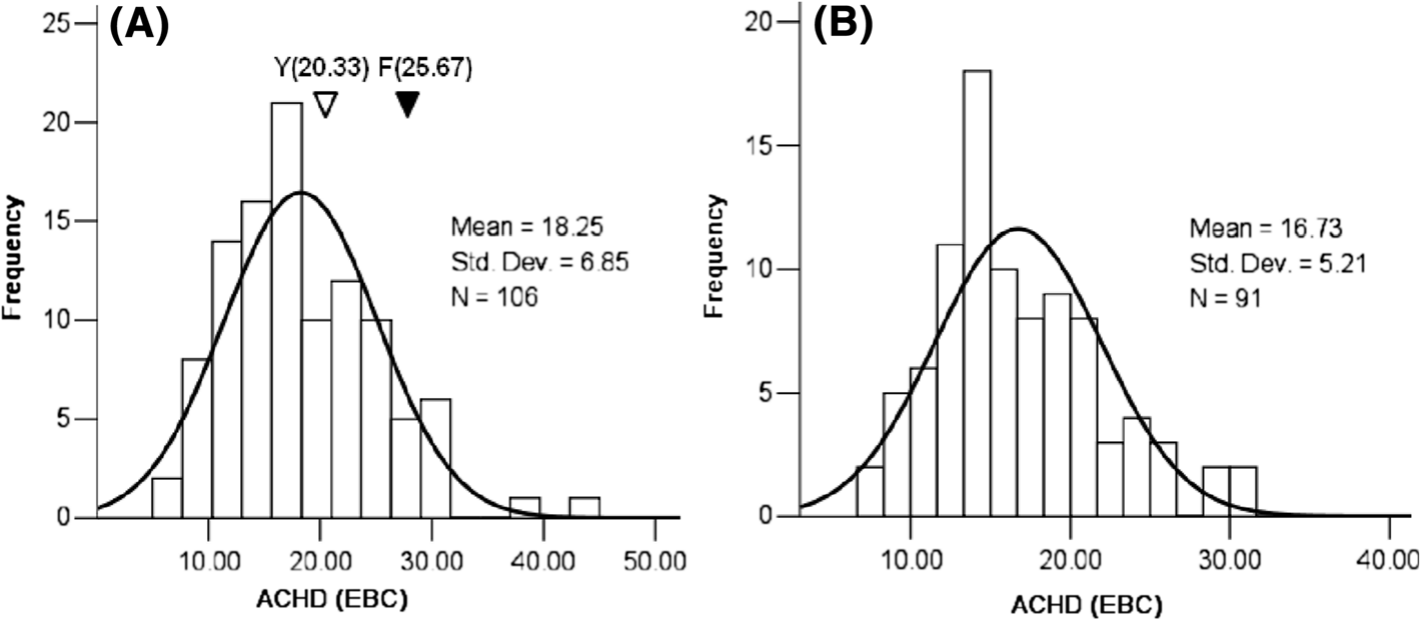
Frequency distribution of alcohol chill haze degree (ACHD) in a Franklin/Yerong DH population. **a**,2009–2010 growing season; **b**, 2010–2011 growing season

In 2009–2010 growing season, ACHD of Yerong was 20.33 EBC, being significantly lower than that of Franklin (25.67 EBC unit, *P* < 0.01). As shown in Fig. 1, there was a large difference in ACHD among the lines of Franklin/Yerong DH population. ACHD showed the normal distribution in the population in the two growing seasons. In 2009–2010 growing season, the mean ACHD of all examined DH lines was 18.25 EBC (Fig. 1). Transgression beyond the parental values could be observed. In 2010–2011 growing season, the mean ACHD of the examined lines was 16.73 EBC. The pairwise correlation coefficient of ACHD between the two years was significantly positive (r = 0.62, *P* < 0.01, Additional file 2: Figure S1).

### Identification of QTLs associated with alcohol chill haze in beer

**Fig. 2:**
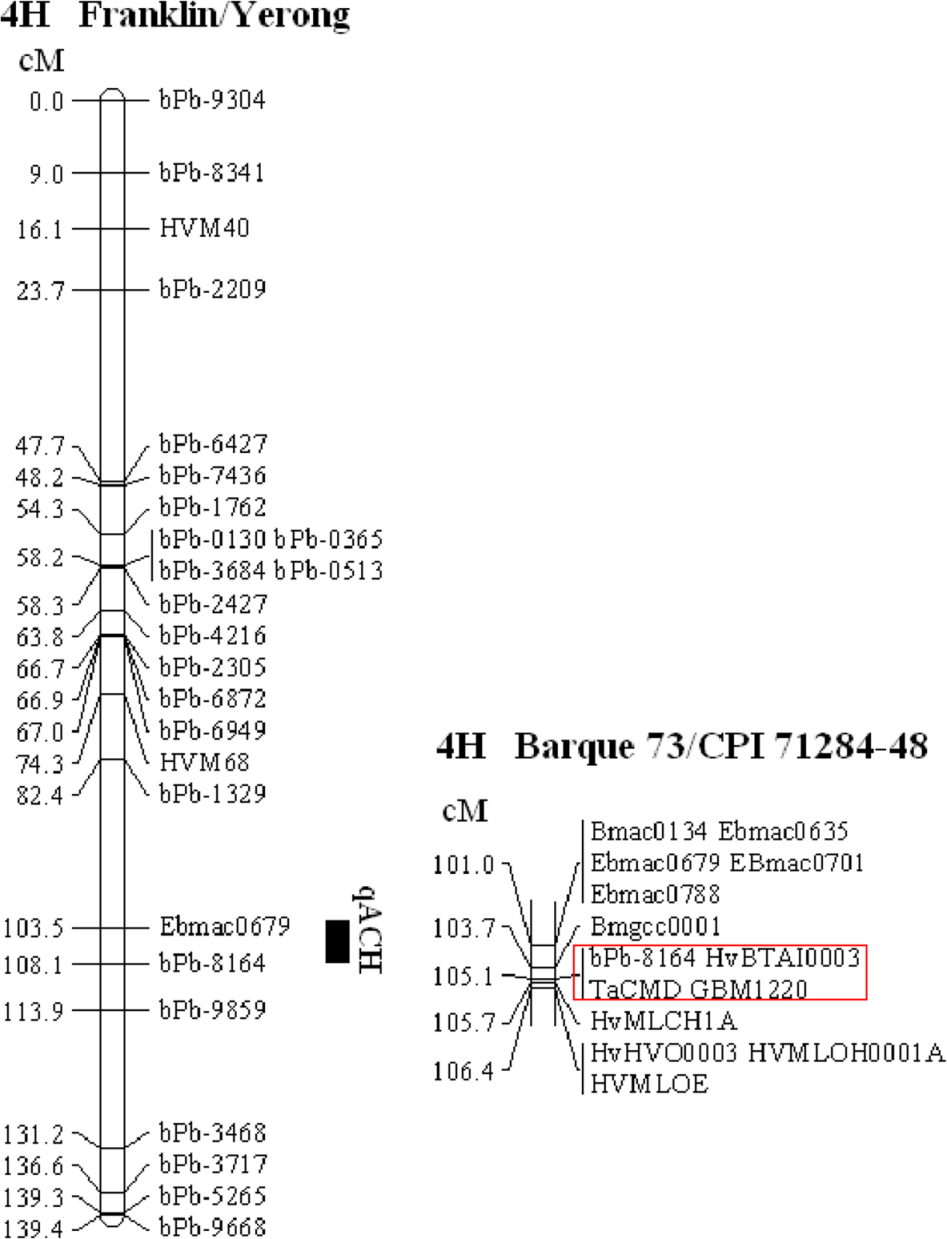
Quantitative trait loci (QTLs) identified for alcohol chill haze in chromosome 4H of Franklin/Yerong population (Li et al. 2008). Part of genetic map of Barque/CPI 71284-48population (Hearnden et al. 2007) was added to the right for comparison

Only one QTL controlling ACHD was found on chromosome 4H in both growing seasons (Table 1, Fig. 2), with the nearest marker being bPb-8164. This QTL could explain around 20 % of the phenotypic variation. The QTL was named as *qACH*,a novel locus associated with beer haze stability.

**Table 1:** QTLs identified for ACHD in aFranklin × Yerong DH population

Year	Chr.	Closest marker	Position (cM)	LOD	R^2^ (%)	Additive	Y* (EBC)	F* (EBC)	QTL
2009–2010	4H	bPb-8164	108.093	3.88	20.3	2.35	14.31	18.73	*qACH*
2010-2011	4H	bPb-8164	108.093	4.85	19.3	3.05	14.87	20.94	*qACH*

*: Y and F indicated that the genotypes of marker bPb-8164belong to the same groups of Yerong and Franklin, respectively. These values were the mean ACHD of each group

The results of QTL analysis showed that the mean ACHD of Yerong’ and Franklin’ groups in the population was 14.31 and 18.73 EBC in 2009–2010 growing season, 14.87 and 20.94 EBC in 2010–2011 growing season, respectively (Table 1, *P < 0.01*). Obviously, ACHD of Yerong-derived genotypes is constantly lower than that of Franklin-derived genotypes.

### Identification of haze active proteins in beer

**Fig. 3:**
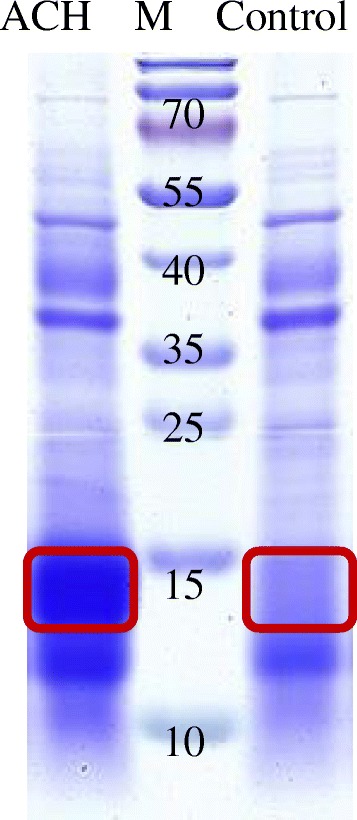
SDS-PAGE analysis of turbid sediment (ACH) and clear supernatant (Control). The protein band in red frame was cut for LC-MS analysis, M indicates protein ladder

The clear supernatant (Control) and haze sediment after alcohol chill test were analyzed by SDS-PAGE (Fig. 3). Many bands, ranging from 35 kDa to 55 kDa for both samples and from 10 kDa to 15 kDa for ACH, were detected. These detected proteins could be derived from both brewing yeast and malt. In addition, there were a few of bands being larger than 55 kDa, indicating that during malting, mashing and brewing, some barley proteins of larger molecular weight are degraded to smaller proteins chemically and proteolytically. The bands ranging from 13 to 15 kDa are particularly interesting, as a polymorphism was observed for the bands rich in ACH, but relatively poor in Control. It may be suggested that the bands showing polymorphism might be the crucial haze active proteins influencing beer haze stability.

The special proteins ranging from 13 kDa to 15 kDa in both Control and ACH were excised and digested by trypsin for LC-MS/MS analysis. After LC-MS identification, corresponding peptide sequences were obtained. For data processing, MASCOT search program was performed at significant threshold of 0.05, so as to provide the amino acid sequences, GI number and scores of the relevant proteins. The results showed that there were 4 kinds of proteins in each sample (Table 2). Trypsin inhibitor CMe precursor (BTI-CMe) and α-amylase/trypsin inhibitor CMd (BATI-CMd) were specifically detected in ACH, indicating that they might be important alcohol chill haze active proteins. Glyceraldehyde-3-phosphate dehydrogenase (GAPDH) and α-amylase/trypsin inhibitor CMb (BATI-CMb) were detected in both ACH and Control. As GAPDH was a constitutively expressed protein in barley and yeast, it should be a common protein in beer, not a haze active protein. In contrast, BATI-CMb was detected in ACH and it is quite similar to BATI-CMd, an identified haze active protein. Hence it could be assumed that BATI-CMb is also an important alcohol chill haze active protein. The current results showed that BTI-CMe, BATI-CMb and BATI-CMd are the crucial haze active proteins in the 13–15 kDa bands.

**Table 2:** LC-MS-identifiedproteins of MW ~ 15 kDa (as showed in red frame of Fig. 3) from ACH and Control

Sample	Protein	GI	MW (kDa)	Score
ACH	Trypsin inhibitor CMe precursor	1405736	16.34	99
	Glyceraldehyde-3-phosphate dehydrogenase	34787348 /151943468	11.04	94 /218
	α-amylase/trypsin inhibitor CMb	585290	17.20	86
	α-amylase/trypsin inhibitor CMd	585291	19.14	68
Control	Glyceraldehyde-3-phosphate dehydrogenase	34787348 /151943468	11.04	88 /236
	α-amylase/trypsin inhibitor CMb	585290	17.20	81
	Pathogenesis-related protein 4	1808651	16.08	60
	α-amylase inhibitor BMAI-1	2506771	16.38	53

Individual ions scores > 46 indicate identity or extensive homology (*p* < 0.05), GI and score with underscore indicate the proteins were yeast original

### Genetic analysis of *qACH* and haze active proteins

**Fig. 4:**
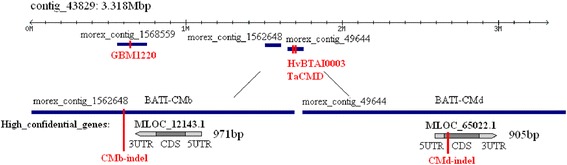
Physical location of *qACH* associated markers, candidate genes (*BATI-CMb*, *BATI-CMd*), and corresponding gene specific markers (CMb-indel, CMd-indel). Words in red color indicatemarkers

The LC-MS identified proteins were firstly searched in NCBI to obtain the corresponding nucleotide sequences, and then the nucleotide sequences were searched in barley genome database (Mayer et al. 2012) to obtain the corresponding gene locations (Table 3). The genetic locations of two haze active proteins, BATI-CMb and BATI-CMd, were at 99.43 cM of chromosome 4H, being similar with the genetic location of *qACH*. Furthermore the two shotgun contigs (morex_contig_1562648, morex_contig_49644) where BATI-CMb and BATI-CMd exist were located at contig_43829 (Fig. 4).

**Table 3:** The chromosome location of the LC-MS identified proteins

Proteins	GI	Contig*	Chr.	cM*
Trypsin inhibitor CMe precursor	1405736	morex_contig_1571056	3H	49.72
α-amylase/trypsin inhibitor CMb	585290	morex_contig_1562648	4H	99.43
α-amylase/trypsin inhibitor CMd	585291	morex_contig_49644	4H	99.43
Glyceraldehyde-3-phosphate dehydrogenase	34787348	morex_contig_1579793	7H	70.54
Pathogenesis-related protein 4	1808651	morex_contig_270946	3H	148.65
α-amylase inhibitor BMAI-1	2506771	morex_contig_113832	2H	141.93

* The information was from barley genome database (Mayer et al. 2012). Words in bold were the information about identified alcohol chill haze active proteins

Because there is no sequence data of DArT marker bpb-8164, three SSR markers closest to it were studied. From GrainGenes database (http://wheat.pw.usda.gov/GG2/index.shtml), GBM1220, HvBTAI0003 and TaCMD were found to be 0 cM distance from bpb-8164 in Barque 73/CPI 71284–48 population (Fig. 2). Moreover, it was found that the physical locations of the above three markers were at morex_contig_1568559, morex_contig_49644 and morex_contig_49644, respectively. These shotgun contigs were all located at contig_43829 (Fig. 4). Therefore it may be concluded that bpb-8164 is located at contig_43829, or the site being very close to this contig.

From the fact that the physical locations of *BATI-CMb* and *BATI-CMd* are very close to the *qACH* associated marker (bpb-8164) (Fig. 4) and that *BATI-CMb* and *BATI-CMd* are alcohol haze active proteins (Fig. 3, Table 2), it may be deduced that *BATI-CMb* and *BATI-CMd* are candidate genes of *qACH*, controlling haze stability of beer.

### Polymorphism of *BATI-CMb* and *BATI-CMd*

The nucleotide and amino acid sequences of *BATI-CMb* and *BATI-CMd* in Yerong (low haze) and Franklin (high haze) were analyzed. According to the reference sequence from barley genome database, 2 sets of primers were designed to amplify *MLOC_12143.1* and *MLOC_65022.1* (encoding BATI-CMb and BATI-CMd). The gene structures were shown in Fig. 4. There was no intron in both genes. After amplification and sequencing, *MLOC_12143.1* and *MLOC_65022.1* of Franklin and Yerong were aligned by software Geneious 4.8.3. The results showed that there were 9 single nucleotide polymorphisms (SNPs) in the coding sequence (CDS) region of *BATI-CMb* between Yerong and Franklin (Additional file 3: Table S2). Seven SNPs were mis-sense mutants among them, causing the changes of 5 amino acids. There were 6 bp deletions and 1 SNP in the CDS region of *BATI-CMd* between Yerong and Franklin (Additional file 4: Table S3). The 6 bp deletions caused loss of 2 amino acids for BATI-CMd in Franklin, indicating the change of amino acid may cause the difference in haze activity (Table 4). Moreover, there were several insertions, deletions and substitutions in the non-coding region of BATI-CMb and BATI-CMd (Additional file 3: Table S2 and Additional file 4: Table S3), which might result in the difference of expression level.

**Table 4:** The difference in amino acid composition of BATI-CMb and BATI-CMd in Franklin and Yerong

	Sequence NO.	Franklin	Yerong
BATI-CMb	18	isoleucine^2^	threonine^1^
	28	glutamic acid^1^	lysine^1^
	57	methionine^2^	threonine^1^
	119	alanine^2^	threonine^1^
	122	phenylalanine^2^	tyrosine^1^
	133	phenylalanine^2^	tyrosine^1^
	140	serine^1^	tryptophan^2^
BATI-CMd	21	-	alanine^2^
	22	-	alanine^2^

1: hydrophilic amino acid, 2: hydrophobic amino acid,-: deletion

### Development of molecular markers

**Fig. 5:**
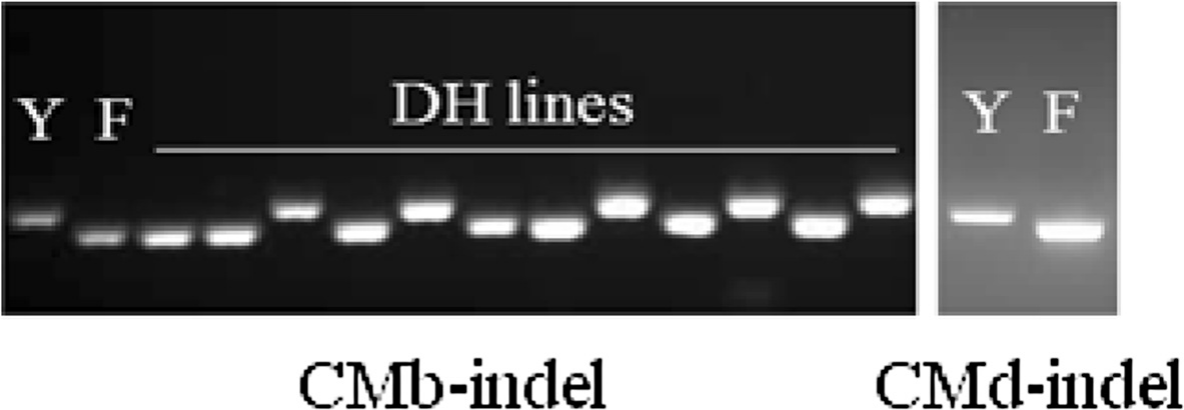
The PCR results of InDel markers screening Franklin, Yerong and their DH lines. Y, Yerong; F, Franklin

Based on the sequence data (Additional file 5), gene specific markers were developed for *BATI-CMb* and *BATI-CMd*. The locations of two markers (CMb-indel and CMd-indel) are shown in Fig. 4. The two markers can clearly distinguish Yerong, Franklin and DH lines (Fig. 5). The PCR products of Yerong and Franklin were 148 bp and 120 bp in the CMb-indel marker, 114 bp and 108 bp in the CMd-indel marker, respectively. After screening the DH population, it was found that the two markers were closely linked. There was no recombinant line in Franklin/Yerong population. So it is hard to determine which haze active protein is more important for haze formation in beer. Combined with initial markers found in Franklin/Yerong DH population, a new genetic map was constructed, and then QTL for alcohol chill haze was analyzed again. As a result, *qACH* was located at the same position, thus confirming that *BATI-CMb* and *BATI-CMd* are indeed the genes controlling beer chill haze.

## Discussion

Evaluation of the difference among malt barley cultivars (genotypes) in beer haze formation and identification of the major factors controlling haze formation are dependent on availability of mini-scale beer preparation in laboratory and the haze-reflecting indicators. In this study, a small barley sample (200 g) of Franklin/Yerong DH population was used to produce beer using micro-malting and micro-brewing according to Cai et al. [16] and Stewart et al. [17] with some modification. In addition, we used ACHD value to indicate the extent of haze formation in beer, since it could predict the colloidal stability and shelf life of beer [18].

A normal distribution of ACHD value among all lines of the DH population indicates that ACH may be controlled by multiple genes. Only one QTL was identified for ACHD, and it can explain about 20 % of the phenotypic variation and has been located on chromosome 4H. In our knowledge, it is the first QTL reported so far to be associated with beer haze stability, and is named as *qACH*. By analysis of proteomics and bioinformatics, a protein, BATI-CMd was detected in ACH. This protein is very similar to an identified haze active protein BATI-CMb [7]. Accordingly, we assumed that BATI-CMd might also be an important alcohol chill haze active protein. Genetic analysis of *qACH* and the potential haze active proteins showed that the physical locations of both *BATI-CMb* and *BATI-CMd* were very close to the *qACH* associated marker (bpb-8164) (Fig. 4), suggesting that both *BATI-CMb* and *BATI-CMd* are critical alcohol haze active proteins controlling haze stability in beer.

Similar to the previously reported haze active protein BATI-CMe [11, 25], BATI-CMb and BATI-CMd belong to chloroform/methanol soluble (CM) proteins. The mechanism for haze formation of BATI-CMb and BATI-CMd could be similar to that of BTI-CMe. The CM proteins belong to the trypsin/α-amylase inhibitor family and make the function in the defense of plants against their bio-aggressors [26, 27]. They are highly expressed during the late stage of seed development and early stage of seed germination in endosperm [28], and are rich in cereal endosperm and heat-stable [29]. During malting and brewing from barley grains, most present heat-stable proteins are disulfide-rich proteins, including trypsin/α-amylase inhibitors [29]. Hence, after malting and brewing, the CM proteins are still abundant and stable in beer. In addition, the CM proteins are rich in cysteine, which is easy to form disulfide bonds and hydrophobic groups. Therefore, the mechanism of haze formation for BATI-CMb and BATI-CMd might be their abundance in beer and easy to form hydrophobic groups. For further investigation, extraction or recombination of these haze active proteins should be highly addressed.

The amino acid sequences differed between the two genotypes (Table 4). In particular for BATI-CMb, there were 5 hydrophobic amino acids in Franklin, whereas there was only a hydrophilic amino acid in Yerong. The difference could be considered as a major cause of the different haze formation in the two genotypes and also suggests the possibility of reducing haze formation in beer through genetic improvement of malt barley cultivars.

## Conclusion

In conclusion, we identified a novel QTL, *qACH* controlling chill haze of beer and two key haze active proteins, BATI-CMb and BATI-CMd. Bioinformatics analysis further suggests that *BATI-CMb* and *BATI-CMd* are the candidate genes associated with beer chill haze. The genotypic difference in haze formation could be attributed to different nucleotide sequence of *BATI-CMb* and *BATI-CMd*.

## Availability of supporting data

The data sets supporting the results of this article are included within the article and its additional files.

## Additional files

Additional file 1: Table S1. The primers involved in cloning and developing molecular makers about *BATI-CMb* and *BATI-CMd.*
Additional file 2: Figure S1. The correlation of ACHD between the two growing years. Additional file 3: Table S2. The SNPs and InDels of *BATI-CMb* (*MLOC_12143.1*) between Franklin and Yerong. Additional file 4: Table S3. The SNPs and InDels of *BATI-CMd* (*MLOC_65022.1*) between Franklin and Yerong. Additional file 5:
**Sequence information of** ***MLOC_12143.1*** **and** ***MLOC_65022.1*** **in Yerong and Franklin.**

## Abbreviations

ACH: Alcohol chill haze
ACHD: Alcohol-chill haze degree
BATI-CMb: CMb component of barley tetrameric α-amylase inhibitor
BATI-CMd: CMd component of barley tetrameric α-amylase inhibitor
BDAI-1: Barley dimeric α-amylase inhibitor
BTI-CMe: Barley trypsin inhibitor CMe precursor
CDS: Coding sequence
CM protein: Chloroform/methanol soluble proteins
DH: Double haploid
EBC: European brewery convention
InDel: Insertion and deletion
LC-MS: High performance liquid chromatography-tandem mass spectrometry
QTL: Quantitative trait loci
SNP: Single nucleotide polymorphism
