# Supplementary material for: Identification of two key genes controlling chill haze stability of beer in barley (Hordeum vulgare L)
Source: BMC Genomics. 2015 Jun 11;16(1):449. doi: 10.1186/s12864-015-1683-1 (PMC4461983; doi:10.1186/s12864-015-1683-1)
Supplement: Additional file 1: Table S1. — The primers involved in cloning and developing molecular makers about BATI-CMb and BATI-CMd. [file 12864_2015_1683_MOESM1_ESM.docx]

Table S1. The primers involved in cloning and developing molecular makers about *BATI-CMb* and *BATI-CMd*

|  |  | BATI-CMb | BATI-CMd |
| --- | --- | --- | --- |
| Cloning | Forward (5'->3') | CMb-F: GCTGGTTTAGCAACAGTCTCAC | CMd-F: ATGCGGCATCAACGCTTCCA |
|  | Reverse (5'->3') | CMb-R: AGTATTTGTCGGTCTGGTATTGA | CMd-R: AGCTAGGCTCCAGACGTGCC |
| Marker | Forward (5'->3') | CMb-InDel-F: AAGCATCTCCGGTGGCGGAT | CMd- InDel-F: GCAAGTCCAGCCGCAGTCTC |
|  | Reverse (5'->3') | CMb- InDel-R: CCTCCTACAGATAACGTCCGGCT | CMd- InDel-R: TGGTCGGAAAAGCCACCCCT |
