# Supplementary figures and images for: Identification of two key genes controlling chill haze stability of beer in barley (Hordeum vulgare L)
Source: BMC Genomics. 2015 Jun 11;16(1):449. doi: 10.1186/s12864-015-1683-1 (PMC4461983; doi:10.1186/s12864-015-1683-1)

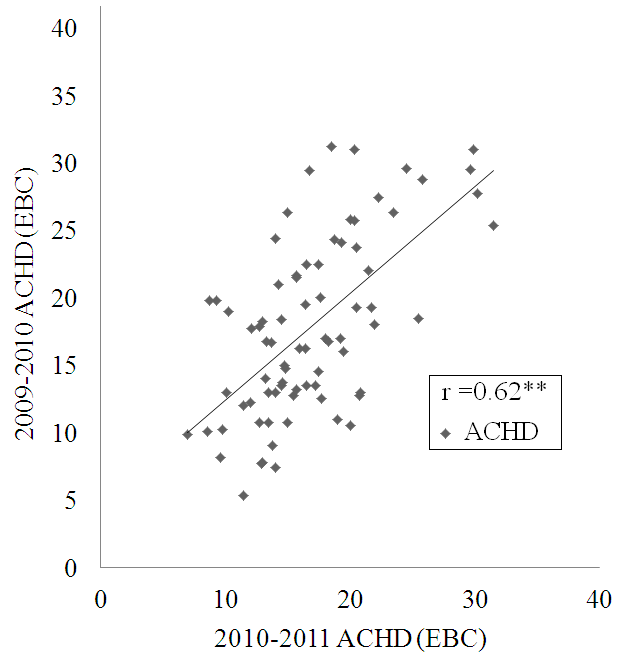


Figure S1. The correlation of ACHD between the two growing years

Supplement: Additional file 2: Figure S1. — The correlation of ACHD between the two growing years. [file 12864_2015_1683_MOESM2_ESM.docx]
