# Supplementary material for: Identification of two key genes controlling chill haze stability of beer in barley (Hordeum vulgare L)
Source: BMC Genomics. 2015 Jun 11;16(1):449. doi: 10.1186/s12864-015-1683-1 (PMC4461983; doi:10.1186/s12864-015-1683-1)
Supplement: Additional file 3: Table S2. — The SNPs and InDels of BATI-CMb (MLOC_12143.1) between Franklin and Yerong. [file 12864_2015_1683_MOESM3_ESM.docx]

Table S2. The SNPs and InDels of *BATI-CMb* (*MLOC_12143.1*) between Franklin and Yerong

| Region | | SNPs/ InDels in *BATI-CMb* | | | | | | | | | | | | | | |
| --- | --- | --- | --- | --- | --- | --- | --- | --- | --- | --- | --- | --- | --- | --- | --- | --- |
| 5'UTR | NO. | 32 | 51 | 161 | 187 |  |  |  |  |  |  |  |  |  |  |  |
|  | Franklin | C | G | C | G |  |  |  |  |  |  |  |  |  |  |  |
|  | Yerong | T | A | A | T |  |  |  |  |  |  |  |  |  |  |  |
| CDS | NO. | **286** | 311 | **315** | **403** | **588** | **598** | **631** | 647 | **652** |  |  |  |  |  |  |
|  | Franklin | **T** | T | **G** | **T** | **G** | **T** | **T** | C | **C** |  |  |  |  |  |  |
|  | Yerong | **C** | C | **A** | **C** | **A** | **A** | **A** | T | **G** |  |  |  |  |  |  |
| 3'UTR | NO. | 666 | 669 | 678 | 679 | 680 | 681 | 682 | 721 | 722 | 733 | 808 | 831 | 832 | 877 | 903 |
|  | Franklin | G | T | C | T | C | C | C | - | - | G | A | T | C | A | G |
|  | Yerong | A | C | A | - | - | - | - | C | G | A | C | A | A | G | A |

Words in bold indicates mis-sense mutation, - indicates deletion
