# Supplementary material for: Identification of two key genes controlling chill haze stability of beer in barley (Hordeum vulgare L)
Source: BMC Genomics. 2015 Jun 11;16(1):449. doi: 10.1186/s12864-015-1683-1 (PMC4461983; doi:10.1186/s12864-015-1683-1)
Supplement: Additional file 4: Table S3. — The SNPs and InDels of BATI-CMd (MLOC_65022.1) between Franklin and Yerong. [file 12864_2015_1683_MOESM4_ESM.docx]

Table S3. The SNPs and InDels of *BATI-CMd* (*MLOC_65022.1*) between Franklin and Yerong

| Region | | SNPs/ InDels in *BATI-CMb* | | | | | | | | | | | | | | | |
| --- | --- | --- | --- | --- | --- | --- | --- | --- | --- | --- | --- | --- | --- | --- | --- | --- | --- |
| 5'UTR | NO. | 22 | 23 |  |  |  |  |  |  |  |  |  |  |  |  |  |  |
|  | Franklin | - | - |  |  |  |  |  |  |  |  |  |  |  |  |  |  |
|  | Yerong | T | G |  |  |  |  |  |  |  |  |  |  |  |  |  |  |
| CDS | NO. | **190** | **191** | **192** | **193** | **194** | **195** | 395 |  |  |  |  |  |  |  |  |  |
|  | Franklin | **-** | **-** | **-** | **-** | **-** | **-** | T |  |  |  |  |  |  |  |  |  |
|  | Yerong | **C** | **C** | **G** | **C** | **T** | **G** | C |  |  |  |  |  |  |  |  |  |
| 3'UTR | NO. | 642 | 698 | 699 | 700 | 701 | 702 | 703 | 711 | 713 | 714 | 715 | 721 | 724 |  |  |  |
|  | Franklin | T | G | C | T | T | G | T | G | G | T | G | A | C |  |  |  |
|  | Yerong | G | - | - | - | - | - | - | C | C | A | A | G | G |  |  |  |
| 3'UTR | NO. | 726 | 730 | 731 | 732 | 733 | 734 | 737 | 738 | 758 | 773 | 854 | 866 | 875 | 892 |  |  |
|  | Franklin | - | G | T | G | A | G | C | C | G | C | - | G | A | C |  |  |
|  | Yerong | C | - | - | - | - | - | - | - | A | T | A | A | C | G |  |  |

Words in bold indicates mis-sense mutation, - indicates deletion
