# Supplementary material for: Identification of two key genes controlling chill haze stability of beer in barley (Hordeum vulgare L)
Source: BMC Genomics. 2015 Jun 11;16(1):449. doi: 10.1186/s12864-015-1683-1 (PMC4461983; doi:10.1186/s12864-015-1683-1)
Supplement: Additional file 5: — Sequence information of MLOC_12143.1 and MLOC_65022.1 in Yerong and Franklin. [file 12864_2015_1683_MOESM5_ESM.docx]

Sequence information of MLOC_12143.1 and MLOC_65022.1 in Yerong and Franklin

**>MLOC_12143.1_Yerong**

GCAAAAGCCAGCTTGCATTCCCCTCCAAATATAGTTCCAACTAACTAATTATGTGCACTCTAGCTCATCATAATCATCATATAACACTACAAATTTAGTTAATTGCGACCAGCTCTGCCTTTCTAACCTTTCTAGGGGTATATATCCATGCAAGTGTGGTACATGTAGCATACACCCACCAGTGCATTGCAAAAAAGAAACTACACCAACGAACTTGGCCTCCATCCAAAAATATGGCGTCCAAGTCCAGCTGCGATCTCCTCTTGGCCGCCGTCCTAGTCTCCACCTTTGCCGCCGTTGCCGCCGTCGGCAGCAAAGATTGCACCCCATGGACGGCTACTCCGATCACTCCACTCCCAAGTTGCCGCGACTATGTGGAACAGCAAGCATGTCGCATCGAAACGCCCGGGCCGCCGTACCTCGCCAAGCAGCAGTGCTGCGGGGAGCTTGCAAACATCCCGCAGCAGTGCCGATGCCAAGCGTTGCGCTTCTTCATGGGGCGGAAGTCTCGTCCCGATCAGAGCGGCCTCATGGAACTCCCCGGGTGCCCTAGGGAGGTGCAGATGGACTTCGTCAGGATACTCGTCACGCCGGGGTACTGCAACTTGACGACCGTTCACAACACTCCATACTGCCTCGCCATGGATGAGTGGCAGTGGAACTAGAGACAATTCTGTAGCTCATGAATAAATAAGCATGTTGCGACCATACATGTGCGTGACATGCATATATACATATATGAGCTCCTCGCGCTCATCATGTGTGGCGCTATGTGTTATATATGGATAAGAATAAAGGGAGTCCTTTTCGGGGTCGCTTCTTAAACAAAACTCAGATCTTCAATTCATGTGTTTTGTGGTCTAAATTATTAGGAAAGTCGAGAAGCATCCAACAGTTAATAAAATTTCTACATTTTTTGTAGCTTTTTTATTTCTTGCCTCTGGCGTGACATCAATACCAGACCGAC

**>MLOC_12143.1_Franklin**

GCAAAAGCCAGCTTGCATTCCCCTCCAAATACAGTTCCAACTAACTAATTGTGTGCACTCTAGCTCATCATAATCATCATATAACACTACAAATTTAGTTAATTGCGACCAGCTCTGCCTTTCTAACCTTTCTAGGGGTATATATCCATGCAAGTGTGGTCCATGTAGCATACACCCACCAGTGCAGTGCAAAAAAGAAACTACACCAACGAACTTGGCCTCCATCCAAAAATATGGCGTCCAAGTCCAGCTGCGATCTCCTCTTGGCCGCCGTCCTAGTCTCCATCTTTGCCGCCGTTGCCGCCGTCGGTAGCGAAGATTGCACCCCATGGACGGCTACTCCGATCACTCCACTCCCAAGTTGCCGCGACTATGTGGAACAGCAAGCATGTCGCATCGAAATGCCCGGGCCGCCGTACCTCGCCAAGCAGCAGTGCTGCGGGGAGCTTGCAAACATCCCGCAGCAGTGCCGATGCCAAGCGTTGCGCTTCTTCATGGGGCGGAAGTCTCGTCCCGATCAGAGCGGCCTCATGGAACTCCCCGGGTGCCCTAGGGAGGTGCAGATGGACTTCGTCAGGATACTCGTCACGCCGGGGTTCTGCAACTTGACGACCGTTCACAACACTCCATTCTGCCTCGCCATGGACGAGTCGCAGTGGAACTAGGGATAATTCTGTCTCCCGCTCATGAATAAATAAGCATGTTGCGACCATACATGTGTGACATGCATGTATACATATATGAGCTCCTCGCGCTCATCATGTGTGGCGCTATGTGTTATATATGGATAAGAATAAAGGGAGTCATTTTCGGGGTCGCTTCTTAAACTCAACTCAGATCTTCAATTCATGTGTTTTGTGGTCTAAATTATTAGAAAAGTCGAGAAGCATCCAACAGTTAATAAAATTTCTACATTTTTTGTAGCTTTTTTATTTCTTGCCTCTGGCGTGACATCAATACCAGACCGAC

**>MLOC_65022.1_Yerong**

GCAAAAGCCAGCTTGCATTCCCCTCCAAATACAGTTCCAACTAACTAATTGTGTGCACTCTAGCTCATCATAATCATCATATAACACTACAAATTTAGTTAATTGCGACCAGCTCTGCCTTTCTAACCTTTCTAGGGGTATATATCCATGCAAGTGTGGTCCATGTAGCATACACCCACCAGTGCAGTGCAAAAAAGAAACTACACCAACGAACTTGGCCTCCATCCAAAAATATGGCGTCCAAGTCCAGCTGCGATCTCCTCTTGGCCGCCGTCCTAGTCTCCATCTTTGCCGCCGTTGCCGCCGTCGGTAGCGAAGATTGCACCCCATGGACGGCTACTCCGATCACTCCACTCCCAAGTTGCCGCGACTATGTGGAACAGCAAGCATGTCGCATCGAAATGCCCGGGCCGCCGTACCTCGCCAAGCAGCAGTGCTGCGGGGAGCTTGCAAACATCCCGCAGCAGTGCCGATGCCAAGCGTTGCGCTTCTTCATGGGGCGGAAGTCTCGTCCCGATCAGAGCGGCCTCATGGAACTCCCCGGGTGCCCTAGGGAGGTGCAGATGGACTTCGTCAGGATACTCGTCACGCCGGGGTTCTGCAACTTGACGACCGTTCACAACACTCCATTCTGCCTCGCCATGGACGAGTCGCAGTGGAACTAGGGATAATTCTGTCTCCCGCTCATGAATAAATAAGCATGTTGCGACCATACATGTGTGACATGCATGTATACATATATGAGCTCCTCGCGCTCATCATGTGTGGCGCTATGTGTTATATATGGATAAGAATAAAGGGAGTCATTTTCGGGGTCGCTTCTTAAACTCAACTCAGATCTTCAATTCATGTGTTTTGTGGTCTAAATTATTAGAAAAGTCGAGAAGCATCCAACAGTTAATAAAATTTCTACATTTTTTGTAGCTTTTTTATTTCTTGCCTCTGGCGTGACATCAATACCAGACCGAC

**>MLOC_65022.1_Franklin**

TTGGTTAGTTGCTACCAACCCCCACCCTCAACTTTTTCTGGGTATATATGCAAGCCTGCGTTGTACATGTAGCATACACTCAAGCAACTACACCAACGAACCTGCAGACTCAGCTAGGACAATATGGCGTGCAAGTCCAGCCGCAGTCTCCTCCTCTTGGCCACCGTCATGGTCTCCGTCTTCGCCGCCGCTGCCGCCGCCGCCACCGACTGCTCCCCAGGGGTGGCTTTTCCGACCAATCTGCTCGGACACTGCCGCGACTATGTGTTACAGCAGACTTGTGCCGTCTTCACTCCCGGGTCGAAGTTACCCGAATGGATGACATCCGCGGAGCTGAACTACCCCGGGCAGCCATACCTCGCCAAGTTGTATTGCTGCCAGGAGCTTGCAGAAATTCCCCAGCAGTGCCGGTGCGAGGCGCTGCGCTACTTCATGGCGTTGCCGGTACCGTCTCAGCCCGTGGACCCGAGCACCGGCAATGTTGGTCAGAGCGGCCTCATGGACCTGCCCGGATGCCCCAGGGAGATGCAACGGGACTTCGTCAGATTACTCGTCGCCCCGGGGCAGTGCAACTTGGCGACCATTCACAACGTTCGATACTGCCCCGCCGTGGAACAGCCGCTGTGGATCTAGTGATGATAAAATCAGTCGTTCGTGAATAAGCATGCATGTTGCGTCCATAGGCGTAGGCTTGTGCGTGTGGTGTGCATGTATGCATATGTGAGCTCCGCACGCTCAACATGTGTGGGCTATCTGCTATGAACGAGAATAAAGAGAACCATTTTGTGGTTCTTTAATTTCAACTCTATCATCTTGTTTTGTCAAAGGCCAAAGGCAGATACAAGTTATTAACCAGGTCCAAACATAATAAAAACAGCTATCACAAAAATAAAG
